# Proximal infolding of an Endurant II endograft for barrel-shaped neck: A rare but treatable complication with proximal cuff and coil embolization

**DOI:** 10.1016/j.jvscit.2026.102377

**Published:** 2026-06-29

**Authors:** Hideyasu Ueda, Hiroki Nakabori, Yoshitaka Yamamoto, Kenji Iino

**Affiliations:** Department of Cardiovascular Surgery, Kanazawa University Hospital, Kanazawa City, Ishikawa, Japan

**Keywords:** Barrel-shaped neck, Coil embolization, Endovascular aneurysm repair, Proximal cuff, Proximal infolding

## Abstract

A 75-year-old man with a 55-mm infrarenal abdominal aortic aneurysm underwent endovascular aortic repair. Computed tomography showed a barrel-shaped neck (26 mm at the lowest renal artery, 31.5 mm at 15 mm, and 30.7 mm at 35 mm distal). A 36-mm Endurant II bifurcated graft was deployed. Completion angiography revealed a low-flow endoleak. Postoperative computed tomography demonstrated proximal graft infoldings with a type IA endoleak, likely due to excessive oversizing. Additional endovascular intervention with coil embolization of the gutter and proximal cuff extension eliminated the endoleak. At 12 months, the sac had shrunk, without recurrence.

Endovascular aortic repair (EVAR) offers lower perioperative mortality than open repair for abdominal aortic aneurysm (AAA).^1^^,^^2^ Endograft infolding/collapse is an uncommon complication, reported predominantly after thoracic EVAR and less commonly after EVAR.^3^, ^4^, ^5^, ^6^ This report describes a case of endograft infolding due to a barrel-shaped neck and its endovascular management with coil embolization of the gutter and proximal cuff extension. Informed consent was obtained from the patient for the publication of this case.

## Case report

We report an asymptomatic 75-year-old man with a 55-mm AAA and a history of hypertension, type 2 diabetes, and previous myocardial infarction treated with coronary stents. He had no relevant family history. His medications included atorvastatin, aspirin, and metformin. Computed tomography angiography (CTA) revealed a 55 mm AAA that extended from the aorta at the inferior mesenteric artery branch to the terminal aortic bifurcation (Fig 1, *A*). Centerline imaging confirmed a barrel-shaped neck that measured 26 mm in diameter at the lowest left renal artery, 31.5 mm at 15 mm, and 30.7 mm at 35 mm distal to the lowest renal artery (Fig 1, *B*).

**Fig 1:**
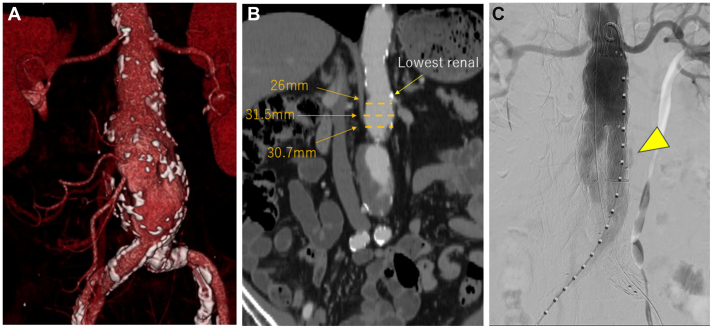
**A,** Reconstruction of computed tomography (CT) angiography image demonstrating infrarenal abdominal aortic aneurysm (AAA) with long infrarenal neck. The image demonstrates minimal calcification or thrombus, with anatomy suitable for endovascular aortic repair (EVAR). **B,** The infrarenal neck was 26 mm in diameter at the lowest renal artery, 31.5 mm at 15 mm, and 30.7 mm at 35 mm distal to the lowest renal artery. **C,** Intraoperative aortography demonstrated endoleak.

-Both open and endovascular repair were discussed with the patient. A joint decision was made to proceed with EVAR. Given the barrel-shaped neck, we sized to the narrow juxtarenal diameter to achieve a durable proximal seal. Fenestrated EVAR was considered but unavailable at our institution. We selected a 36-mm Endurant II main body. This choice resulted in 38% nominal oversizing immediately distal to the lowest renal artery, with 14% oversizing at 15 mm distal and 17% at 35 mm distal. This degree of juxtarenal oversizing is uncommon for cylindrical necks; it was chosen to prioritize juxtarenal sealing, as the bulbous segment was not expected to be the primary sealing zone. We avoided a smaller main body given concern for inadequate juxtarenal seal in this hostile neck.

### The first operative procedure

Under general anesthesia, bilateral surgical femoral access was obtained, and a bifurcated 36-mm main body endoprosthesis (Endurant IIs, Medtronic) was inserted. The repair was extended using a 16 mm iliac extension limb (GORE EXCLUDER Endovascular Graft, L. Gore & Associates). Balloon dilation was performed as a standard completion maneuver to optimize proximal apposition and sealing, using a 37-mm balloon catheter (Cook Medical). We did not upsize to a larger molding balloon because additional radial force was unnecessary and could increase aortic injury risk. Completion aortography showed a low flow, diffuse blush without a focal proximal jet; given the postmolding appearance and no discrete attachment-site leak, we favored transient graft porosity (type IV endoleak) (Fig 1, *C*). Since Endurant's porous multifilament polyester may allow some transfabric movement of contrast in the heparinized patient, we considered this a possible type IV endoleak and terminated the case. Retrospectively, this contrast blush may have been the first harbinger of a more sinister endoleak at the proximal seal. Routine CTA obtained on postoperative day 7 before discharge demonstrated proximal infolding with a type IA endoleak (Fig 2, *A* and *B*), which was further characterized by color Doppler ultrasound study (Fig 2, *C* and *D*). Color Doppler ultrasound guidance served as a noninvasive method to further characterize the suspected proximal endoleak and to establish a baseline for continued monitoring. Given the low-flow endoleak and clinical stability, we counseled the patient, and he chose scheduled elective reintervention. He was discharged the next day and readmitted for planned secondary intervention 1 month later.

**Fig 2:**
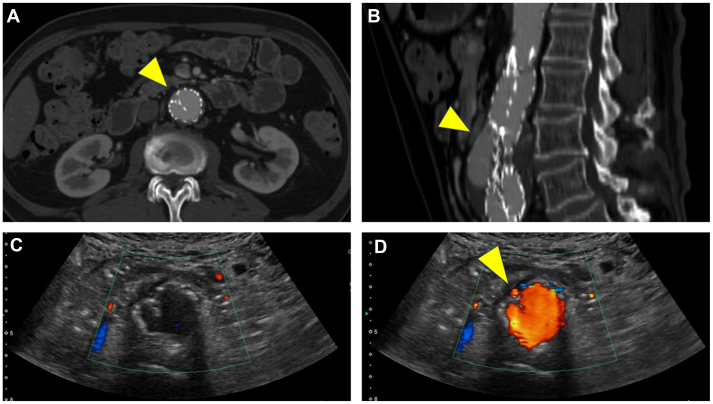
**A,** Axial arterial-phase images of the postoperative computed tomography scan revealed the infolding of the endograft. **B,** Sagittal view demonstrated type 1A endoleak. **C** and **D,** The abdominal ultrasound study immediately before re-EVAR showed no change in the infolding status, and color Doppler imaging also revealed a type 1A endoleak.

### The second operative procedure

Under general anesthesia, unilateral percutaneous femoral access was achieved. Following a coiled embolization of the gutter (AZUR soft 3D, Terumo Medical Corporation): 4 mm × 15 cm (6 coils), aortic cuff was deployed (GORE EXCLUDER Endovascular Graft, L. Gore & Associates) 4.5 cm × 32 mm (Fig 3). Because of irregular neck geometry and intentional cuff downsizing, we added gutter coil embolization to promote thrombosis and reduce persistent type IA endoleak. The postoperative course was uneventful. CTA performed immediately before discharge revealed no endoleak (Fig 4, *A*-*C*). The expansion of the aortic cuff was also satisfactory in areas where central coil embolization was performed (Fig 4, *B*). At 12 months of follow-up, the patient had no other complications, and on follow-up CT there was satisfactory sac contraction to 49 mm (Fig 4, *D*). The patient will be enrolled in close post-EVAR surveillance to monitor for recurrence of the endoleak.

**Fig 3:**
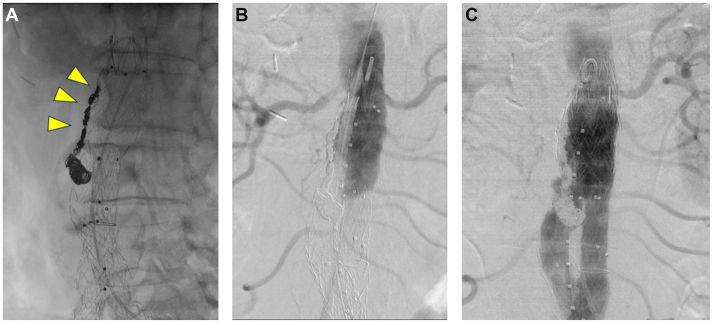
**A,** Selected image from the second endovascular treatment after coil embolization. **B,** Before the deployment of the aortic cuff. **C,** No endoleak was detected after aortic cuff deployment.

**Fig 4:**
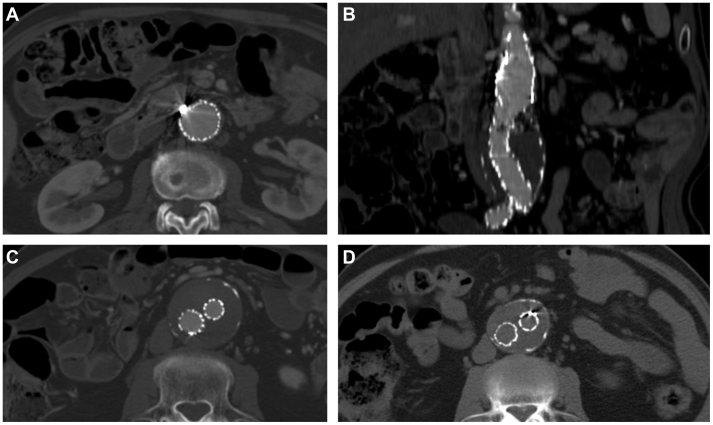
**A**-**C,** The postoperative computed tomography (CT) image shows adequate expansion of the proximal endograft and disappearance of the endoleak. **D,** The 1-year postoperative CT image shows shrinkage of the sac.

## Discussion

Long-term evidence supports the durability of EVAR when performed according to the manufacturer's instructions for use. In ENGAGE registry, Endurant achieved 94.7% 10-year freedom from aneurysm-related mortality and 64.1% sac regression, underscoring the importance of appropriate selection, accurate sizing, and a durable proximal seal.^2^^,^^7^ Against this background, proximal infolding is clinically meaningful because it may undermine fixation and convert an otherwise instructions for use -compliant repair into one at risk of late-type IA endoleak, migration, and reintervention.^5^^,^^6^^,^^8^

In this case, we consider the mechanism to be multifactorial, with disproportionate effective oversizing across a noncylindrical, barrel-shaped proximal neck being a key contributor. In this anatomy, sizing is dictated by a narrow juxtarenal diameter to achieve a seal, whereas the adjacent bulbous segment may exhibit fabric redundancy, local pleating, and uneven wall apposition. Excessive oversizing is associated with adverse EVAR outcomes, and experimental/CT-correlative data suggest it reduces barb penetration and early fixation, predisposing to folding/infolding.^9^, ^10^, ^11^ In addition, complex proximal neck anatomy (eg, barrel-shaped necks) is associated with higher endoleak rates, even when nominal neck length is adequate.^8^^,^^12^ Accordingly, nonuniform effective oversizing within the bulbous segment could plausibly contribute to infolding in our patient.

Oversizing thresholds should not be interpreted universally across devices and clinical contexts. The Endurant-based series in selected hostile neck configurations and chimney EVAR suggest that greater oversizing may be used to enhance sealing in specific scenarios, whereas patients with wide infrarenal necks, where adequate oversizing is inherently limited, appear to have higher rates of proximal failure, implying that “insufficient” oversizing may be the dominant risk factor in some subgroups.^8^^,^^12^^,^^13^ Overall, oversizing should be tailored using multilevel measurements and neck/sealing context—not a single cutoff.

For secondary interventions, balloon re-expansion is a reasonable first maneuver; however, persistent recoil suggests a mechanically stable-folded configuration. Previous reports indicated that proximal infolding/collapse can be corrected endovascularly with an additional proximal cuff and/or structural reinforcement.^4^, ^5^, ^6^ In our case, after ballooning, the 36-mm main body repeatedly reinflected, prompting proximal cuff extension; we downsized to a 32-mm cuff to better match the native aorta and reduce recurrent infolding. Adjunctive embolization was considered in selected cases when residual type IA risk is anticipated.^14^^,^^15^ Adjuncts include balloon-expandable stent reinforcement and, when appropriate, endosuture fixation; however, EndoAnchor (Heli-FX EndoAnchor System; Medtronic) is not approved in Japan.^4^^,^^16^

This case study yields three conclusions. First, early postoperative CTA helps detect proximal malapposition in patients with challenging anatomy.^5^^,^^6^^,^^17^ Second, in barrel-shaped necks, measure oversizing at multiple levels and avoid aggressive upsizing, as effective oversizing may be disproportionate in the bulbous segment.^9^^,^^18^ Therefore, assess the oversizing level-by-level, as a single juxtarenal measurement can overestimate effective oversizing in the bulbous segment. Third, early sac regression after reintervention suggests restored sealing; however, durability after cuff extension with adjunctive embolization remains uncertain, warranting lifelong imaging surveillance.^7^

This case has two limitations. First, a multiangle completion angiography was not performed, precluding definitive exclusion of type IA endoleak. Second, the risk of infolding may have been mitigated by using a 32-mm body.

## Funding

None.

## Declaration of generative AI and AI-assisted technologies in the writing process

During the preparation of this work, the authors used ChatGPT (OpenAI) to improve the readability and language of the manuscript. After using this tool, the authors reviewed and edited the content as needed, and in April 2026, they took full responsibility for the content of the publication.

## Disclosures

None.
